# Auxiliary Segmentation Method of Osteosarcoma in MRI Images Based on Denoising and Local Enhancement

**DOI:** 10.3390/healthcare10081468

**Published:** 2022-08-04

**Authors:** Luna Wang, Liao Yu, Jun Zhu, Haoyu Tang, Fangfang Gou, Jia Wu

**Affiliations:** 1 School of Computer Science and Engineering, Central South University, Changsha 410083, China; 2 The First People’s Hospital of Huaihua, Huaihua 418099, China; 3 Collaborative Innovation Center for Medical Artificial Intelligence and Big Data Decision Making Assistance, Hunan University of Medicine, Changsha 410036, China; 4 Research Center for Artificial Intelligence, Monash University, Clayton, Melbourne, VIC 3800, Australia

**Keywords:** image segmentation, machine learning, non-parameter, localization, enhancement, denoising, CNN, 68T01

## Abstract

Osteosarcoma is a bone tumor which is malignant. There are many difficulties when doctors manually identify patients’ MRI images to complete the diagnosis. The osteosarcoma in MRI images is very complex, making its recognition and segmentation resource-consuming. Automatic osteosarcoma area segmentation can solve these problems to a certain extent. However, existing studies usually fail to balance segmentation accuracy and efficiency. They are either sensitive to noise with low accuracy or time-consuming. So we propose an auxiliary segmentation method based on denoising and local enhancement. The method first optimizes the osteosarcoma images, including removing noise using the Edge Enhancement based Transformer for Medical Image Denoising (Eformer) and using a non-parameter method to localize and enhance the tumor region in MRI images. Osteosarcoma was then segmented by Deep Feature Aggregation for Real-Time Semantic Segmentation (DFANet). Our method achieves impressive segmentation accuracy. Moreover, it is efficient in both time and space. It can provide information about the location and extent of the osteosarcoma as a basis for further diagnosis.

## 1. Introduction

Osteosarcoma is a kind of malignant bone tumor [1], accounting for 20% to 45% of the total malignant bone tumors, with a high incidence rate. The disease is locally aggressive, develops rapidly, and has a high metastasis rate [2,3,4,5]. Due to the chemotherapy resistance of osteosarcoma and the high recurrence rate of the disease, its overall prognosis is still unsatisfactory [6,7,8,9]. At present, osteosarcoma still has high morbidity and the mortality rate, about 35% of the patients face amputation [10], and survival rate is about 20% [11]. Early detection and localization of disease before surgery or treatment can improve overall survival and reduce amputation rates [12].

Among imaging methods used in order to achieve the clinical evaluation of osteosarcoma, MRI has good soft-tissue contrast and is sensitive to osteosarcoma, which can detect abnormal signals in the early stage of lesions. Its multi-parameter and multi-planar slicing capabilities can display the location and extent of lesions [13,14]. Therefore, MRI is often used to diagnose osteosarcoma. After the clinical acquisition of patients’ MRI images, diagnosis requires tumor identification and delineation [12].

In most developing countries, the diagnosis of osteosarcoma is difficult due to economic backwardness, shortage of medical resources and lack of experienced doctors [15]. Conventional methods of osteosarcoma diagnosis rely on manual tasks. One patient will generate more than 600 MRI images at a time [16], which is a huge amount of data. However, only about 3% of these data are useful, leading to a heavy workload for doctors and inefficiency in diagnosis. The doctor’s manually produced magnetic resonance imaging (MRI) suffers from subjectivity and fatigue limitations. Doctor’s average manual segmentation accuracy is about 90%. For inexperienced doctors, the accuracy of the judgment is only about 85% due to their own subjective judgments or mistakes [17]. The significant differences in the location, extent and shape of osteosarcoma in different patients lead to complex MRI images. Moreover, the tumor area is difficult to identify because of the uneven internal grayscale and texture features. Imaging will also affect visual effects [18]. Due to the high heterogeneity of osteosarcoma, the formed osteoid is indistinguishable, and the blurring of edges caused by partial volume effects in structural MRI reduces segmentation accuracy.

However, the identification and segmentation of osteosarcoma lesions is necessary. It is the basis for further quantitative analysis or tissue classification. Segmentation is usually achieved by drawing a region of interest (ROI) within the tumor margin [19,20,21]. Compared to manual segmentation, automated methods are generally faster, more objective, and provide more accurate results [22,23]. Therefore, automatic segmentation techniques for osteosarcoma are needed clinically. In recent years, artificial intelligence-assisted segmentation methodologies of osteosarcoma images have also been developed, including fuzzy connectivity [17], region growing, unsupervised clustering methods [24], supervised machine learning methods, etc. [25,26,27]. Using computer-aided diagnosis technology and artificial intelligence systems can ease the problems, including the shortage of medical resources, serious imbalance of doctor-patient ratio, and lack of professional doctors in developing countries.

However, it has been reported that the segmentation accuracy of osteosarcoma is generally 38–89% [12]. In the existing studies on osteosarcoma MRI image segmentation, cluster-based methods are computationally efficient but are sensitive to noise with low accuracy. Learning-based segmentation methods cannot balance accuracy and segmentation efficiency. CNN-based segmentation methods are more accurate but time-consuming and memory-consuming. In order to find an osteosarcoma image segmentation method with high accuracy, high efficiency, high degree of automation, and good reproducibility, we propose a method based on denoising and tumor localization and enhancement.

Our method aims to achieve automatic segmentation of osteosarcoma target regions in MRI images so that doctors can accurately and quickly analyze the patient’s condition on this basis. The method firstly preprocesses the osteosarcoma MRI image dataset, uses the Eformer model to remove noise, and then uses non-parametric localization and enhancement methods to dispose of the tumor area, making it clearer and easier to identify. The osteosarcoma region was then segmented using DFANet. DFANet is a lightweight real-time semantic segmentation network that handles image segmentation with fast speed, high efficiency, considerable accuracy and low memory consumption.

Image data preprocessing, including denoising and local enhancement, can improve the accuracy of segmentation, and the use of real-time semantic segmentation network can improve the calculation speed. Therefore, our method also improves efficiency on the basis of ensuring accuracy.

Using the method in this paper to process MRI images, the location and extent of osteosarcoma can be obtained, providing radiologists with intuitive features of osteosarcoma as a reliable basis for subsequent diagnosis and analysis. At the same time, our method can save resources and reduce the cost of osteosarcoma image segmentation processing. This can alleviate the problems of low medical levels and shortage of medical resources in developing countries. It is suitable for clinical use and promotion.

The contributions are as follows:

1. This study proposes an auxiliary segmentation method of osteosarcoma in MRI images based on denoising and local enhancement, improving the accuracy and speed of segmentation and reducing resource consumption.
2. We use the medical denoising model Eformer to remove noise and then localize and enhance the osteosarcoma region in MRI images. After preprocessing, the tumor region in the MRI image will be clearer and the boundary can be enhanced. Finally, an efficient and accurate network DFANet is used to segment osteosarcoma in MRI images.

## 2. Related Works

In the diagnosis of osteosarcoma, using computer-aided diagnosis technology and an artificial intelligence system as auxiliary is particularly important. Current methods for segmenting tumor regions from images can be broadly divided into three categories [28].

The first type of method is based on clustering or clustering. Mandava et al. proposed a spatial fuzzy clustering method based on a multi-criteria optimization method, which considers two criteria of spatial information and intensity based on fuzzy C-means clustering (FCM) [29]. Mohamed Nasor et al. presented a technique using K-means clustering, etc., to complete segmentation [18]. EBK et al. created an automated segmentation system to measure RECIST using SLIC-S and FCM [30]. However, these methods are sensitive to initial noise and can only deal with images with simple structure and ordered texture due to the lack of object priors.

The second type is the traditional learning-based approach. Frangee et al. proposed a method using a supervised cascaded feedforward neural network to complete osteosarcoma segmentation in dynamic perfusion MRI images, training a two-stage cascade classifier model with multi-scale spatial features generated by a pharmacokinetic model [31]. The overall segmentation accuracy is 38–78%. Glass et al. segmented MRI images by a hybrid neural network and then used a multi-layer BPNN to complete classification [32]. Chen et al. used Zernike moment and SVM to segment osteosarcoma in T1-weighted image (TIWI) [33]. The limitation of these methods is that they need to compute a large quantity of features such as texture and wavelet features to train a classifier, which can be slow, time-consuming, and memory-intensive. However, reducing dimensionality [34] or selecting a feature [35] to reduce improvement can result in low accuracy [36]. Therefore, these methods are not effective when the number of osteosarcoma images is large because of handcrafted features and they cannot extract target osteosarcoma tumor regions with complex structures and disordered textures.

The last type of method is based on CNN. Zhang et al. used a multi-supervised residual network (MSRN)-based method [37]. Wu et al. [13] use the Mean Teacher algorithm to optimize the dataset, and the obtained noisy data were subjected to the second round of training. Finally, SepUNet and CRF were used to segment osteosarcoma lesions. Barzekar et al. [38] use CNNs as feature extractors to achieve malignant and benign tumors classification of images. Anisuzzaman et al. used computer-aided detection (CAD) and diagnosis (CADx) to detect osteosarcoma [39].

Based on deep architecture, various CNN-based studies have been used for tumor segmentation in images [40]. CNN operates on patches using kernels without the need to extract handcrafted features, which can significantly improve segmentation accuracy [41]. However, overlapping patches always cause redundancy [42], so these methods are too time-consuming and memory-intensive. The improved fully convolutional network (FCN) based segmentation task achieved good results but failed to identify some smaller object regions [42,43].

## 3. Methods

In the diagnosis of osteosarcoma from patients’ MRI images, there are many difficulties in the traditional manual identification by doctors. Due to a large amount of MRI image data but few useful images generated by patients, the doctors have a large workload and low work efficiency [44,45,46]. The results produced by experience and subjective assessment may also be inaccurate. In the situation of developing countries, because of economic backwardness, shortage of medical resources, and lack of equipment and professionals, early diagnosis of osteosarcoma is more difficult [13].

**Figure 1:**
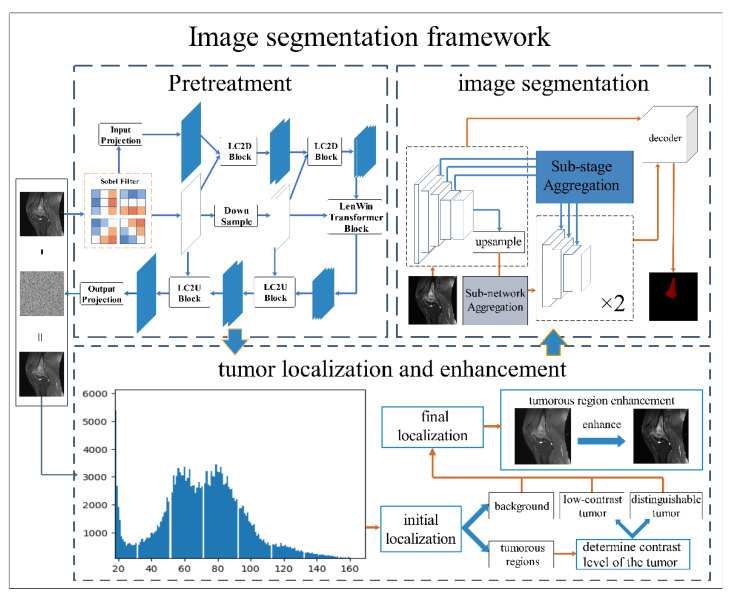
The overall structure of the method.

Segmenting and delineating osteosarcoma in MRI images can assist doctors in determining important information such as the location and extent of lesions [45]. Automated segmentation can reduce labor costs and time costs. It also improves the reliability of segmentation results to a certain extent. In order to further improve the accuracy and efficiency of segmentation, this paper proposes an auxiliary segmentation method of osteosarcoma in MRI images based on denoising and local enhancement. The overall structure of the method is shown in Figure 1.

The method begins with preprocessing the osteosarcoma image dataset, including denoising and tumor localization and enhancement, because MRI images usually have noise affecting the segmentation accuracy. Moreover, the high heterogeneity of osteosarcoma can lead to blurred edges. Therefore, we use the Eformer model to remove noise from MRI images and perform edge enhancement. Afterward, we used non-parametric localization and enhancement methods to localize and enhance the tumor area so that the shape of osteosarcoma is clearer and easier to identify. Osteosarcoma in MRI images was then segmented using DFANet. DFANet is a lightweight network that handles image segmentation quickly and efficiently. We finally obtain the location and extent of osteosarcoma, which provides a more accurate and reliable basis for the subsequent diagnosis and analysis.

The following content is divided into three parts. Firstly, collected MRI images are preprocessed to improve the accuracy and robustness of segmentation and enhance the model’s generalization ability. Section 3.1 shows using Eformer to remove noise in MRI images and perform osteosarcoma edge enhancement. In Section 3.2, we locate and clarify tumor regions, achieving a more efficient segmentation process. Section 3.3 introduces the process of segmenting the preprocessed images using DFANet.

The symbols used in this chapter and their explanations are shown in Table 1.

### 3.1. Remove Noise

**Figure 2:**
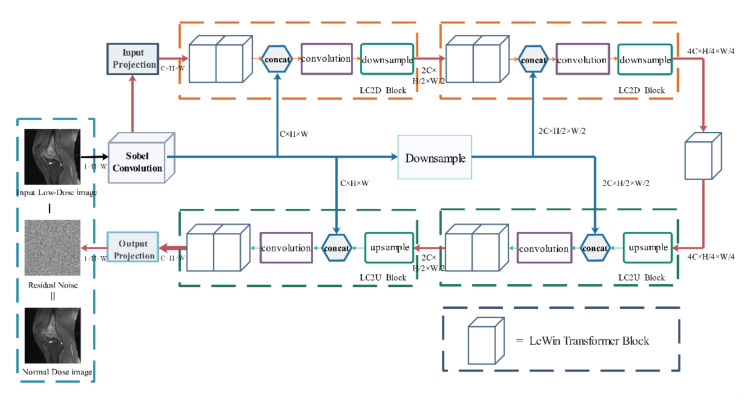
Eformer model structure diagram.

Because the distribution density of osteosarcoma in MRI images is not uniform, the brightness between images is different, and there is much noise in MRI images, which will lead to overfitting of the model. Artifacts caused by partial volume effects and edge blurring caused by high osteosarcoma heterogeneity can affect the subsequent segmentation accuracy. In order to address these issues, we adopt Eformer [47]. Using the Eformer process can effectively remove noise in the MRI images and enhance the edge of the tumor region, improving the accuracy of subsequent segmentation. It utilizes transformer blocks to build an encoder-decoder network. Window-based self-attention is used to reduce computational requirements and effort. Furthermore, Eformer connects the learnable Sobel-Feldman operator to the middle layer of the architecture to enhance the edge and improve the denoising performance. Its model structure is shown in Figure 2.

After inputting the original osteosarcoma MRI image I, the osteosarcoma edge enhancement feature map S(I) is first generated by Sobel Filter and then activated by GeLU [48]. After Sobel Filter post-processing, the feature map of the entire image will also be obtained, and the number of channels has changed. In each LC2D block of encoding stage, the feature map is first processed using LeWin transformer blocks, then concatenated with S(I), and processed by a convolutional layer. Finally, the feature map and S(I) are down-sampled and encoded. The encoded feature map is passed to another LeWin Transformer block for processing. During decoding, the LC2U block up-samples the feature map and then passes it through the convolution block after being concatenated with the previously generated edge feature map S(I). Finally, they are passed to LeWin transformer blocks. The final part of decoding uses a single-layer convolution module output projection. After this process, the noise in the MRI image is removed. Moreover, the edge of the osteosarcoma is enhanced.

The denoising principle of our model is to find out the clean images that may exist in the image through the process of residual learning, and remove them from the original image, and finally obtain the residual, that is, the noise distribution in the image. After the noise distribution is obtained, it is only necessary to remove the noise distribution from the original image to complete denoising.

**Figure 3:**
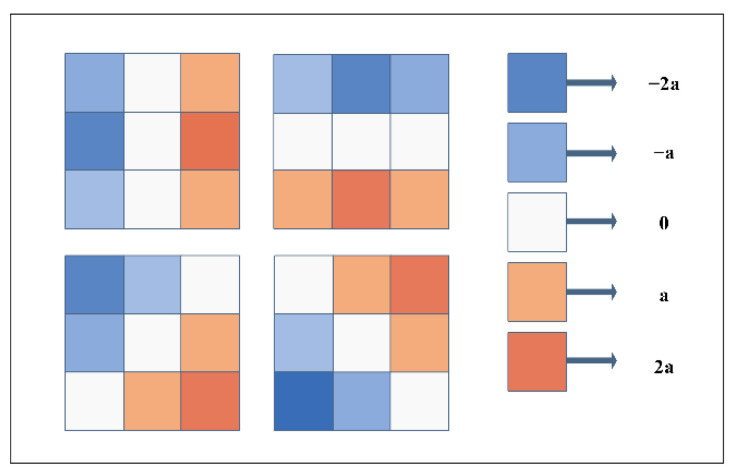
Schematic diagram of Sobel filter.

Sobel-Feldman operator: The Sobel operator is a classic edge detection algorithm [49]. Eformer uses its expanded version, including the diagonal direction, not only the horizontal and vertical directions. The four filters used are shown in Figure 3. The edge enhancement feature map is used many times in the whole network. It is repeatedly cascaded with the image feature map, which can enhance the edge features to the greatest extent and solve the blurred edge of osteosarcoma in images.

Transformer-based encoder-decoder: Both the LC2D and the LC2U use LeWin transformer containing a local enhancement window (LeWin) to process convolutional feature maps. LeWin consists of a low-resolution feature map. Equation (1) is the calculation expression, where *LN* represents layer normalization. $P_{n}$. is the output of *W-MSA* block. $Q_{n}$ is the output of *LeFF* block.

(1) $$\begin{array}{l} P_{n}=W-MSA\left(LN\left(Q_{n-1}\right)\right)+Q_{n-1}\\ Q_{n}=LeFF\left(LN\left(P_{n}\right)\right)+P_{n} \end{array}$$

**Figure 4:**
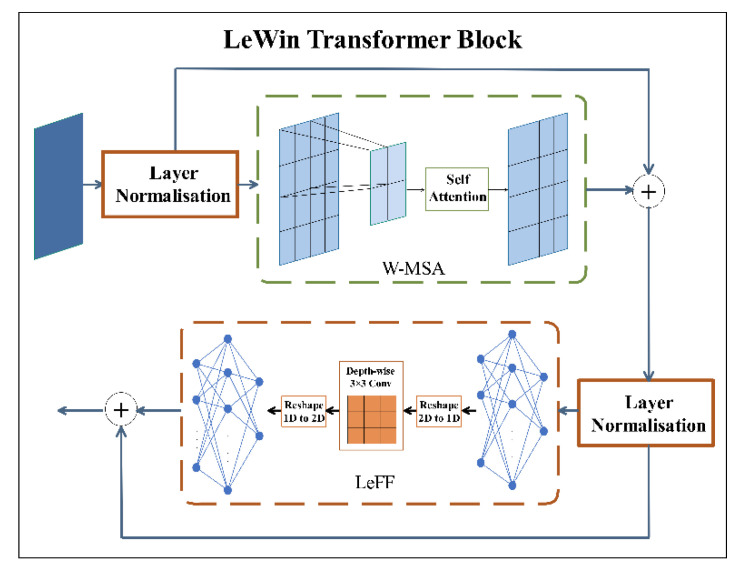
LeWin Transformer Block structure diagram.

We can see the LeWin block’s structure in Figure 4. The feature map is normalized by a Layer Normalization and then passed to *W-MSA*, where the two-dimensional feature map is decomposed into non-overlapping windows, and then the flattened features of each window are performed self-attention. At last it concatenates all outputs and linearly projects the final result. It is passed to locally enhanced feedforward network (*LeFF*) through a Layer Normalization. In *LeFF* block, the image patch is managed through a linear projection layer and a 3 × 3 depth-wise convolutional layer.

Down-sampling & up-sampling: The Eformer down-sampling uses strided convolutions. Up-sampling adopts transpose convolution [50], which can reconstruct the spatial structure. To avoid uneven overlap, the size of the convolution kernel should generally be divisible by the step size, so the size of the convolution kernel of 4 × 4 is selected for transpose convolution, and the step size is set to 2.

Residual Learning: This aims to implicitly remove potentially clean images in hidden layers of MRI images. For example, an MRI image of osteosarcoma that contains noise is t=c+r. Eformer‘s approach is to train a network that can learn this residual mapping $R\left(t\right)=r$, thereby estimating the noise distribution r in the image, and then the denoised image can be obtained through c=t−r.

Optimization: Multiple loss functions are used. First, the Mean Squared Error (MSE) loss function is used to estimate the pixel distance between the actual output and the clean image as in Equation (2). The clean image here refers to the corresponding low-noise image.

(2) $$L_{\mathrm{mse}}=\frac{1}{N}\overset{N}{\underset{i=1}{\sum }}{\left\|\left(t_{i}-R\left(t_{i}\right)\right)-c_{i}\right\|}^{2}$$

However, the MSE loss function easily causes image artifacts such as over-smoothing and blurring, which is very unfavorable for the denoising of osteosarcoma MRI images, so the Multi-scale Perceptual (MSP) [51] loss function used in ResNet is added, as in Equation (3).

(3) $$L_{msp}=\frac{1}{NM}{\sum_{i=1}^{N}\sum_{s=1}^{M}\left\|\phi_{s}\left(t_{i}-R\left(t_{i}\right),\hat{\theta }\right)-\phi_{s}\left(c_{i},\hat{\theta }\right)\right\|}^{2}$$

Among them, ϕ uses ResNet-50 to extract features (but the pooling layer is removed, only the convolutional layer parameters are kept, and the pre-trained model on ImageNet is used). c and $t-R\left(t\right)$ should be processed by the feature extractor and produce the perceptual loss. The final loss function combines the above two loss functions, as shown in the following formula, where the two λ are predefined numbers.

(4) $$L_{final}=\lambda_{mse}L_{mse}+\lambda_{msp}L_{msp}$$

In this way, the combination of perceived loss MSP and mean square error MSE can not only process the overall structural information, but also process the pixel-by-pixel similarity, so as to obtain more accurate processing result, improve the effect of denoising and minimize the loss of important information from the original MRI image of osteosarcoma. After Eformer model processing, we obtained the image denoising and edge enhancement.

### 3.2. Tumor Localization and Enhancement Methods

Since the osteosarcoma region’s location, shape and structure varied greatly, it is difficult to identify in the image. Therefore, non-parametric tumor localization and enhancement methods were used to locate and clarify the tumor region [52] in order to find more accurate targets for subsequent segmentation. Firstly, the frequency distribution histogram of intensity value in MRI image was used to distinguish background and tumor region, and the zero intensity value was dislodged. We calculated the frequency of intensity value by Formula (5). j  represents the range from 0 to the maximum intensity value σ in the image. $n_{j}$ is the frequency distribution value of the *j*th intensity in the MRI image to be calculated. $I_{j}$ is the image. x and y represent the coordinates of the points in the image.

(5) $$n_{j}=\sum_{j=1}^{\sigma }I_{j}\;and\;I\left(x,y\right)\in \left[0,\sigma \right]$$

**Figure 5:**
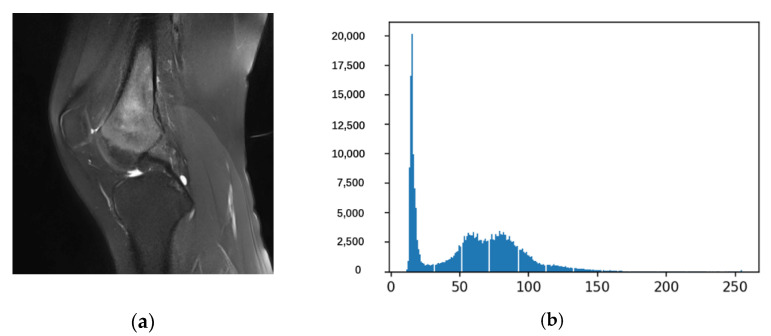
The frequency distribution histogram.

After the above calculation, we can draw the frequency distribution histogram such as Figure 5. Figure 5a is an image that requires osteosarcoma localization and enhancement, and Figure 5b is a histogram of the entire corresponding frequency distribution.

Then, the initial non-parametric threshold $\theta_{}$ (average of the frequency of the intensity values) is determined by the frequency of the intensity values. Its calculation method is shown in Equation (6).

(6) $$\theta_{}=\frac{1}{\sigma }\sum_{j=1}^{\sigma }n_{j}$$

In MRI images of osteosarcoma, the areas with infrequent intensity values represent the background, while the areas with the most frequent intensity values represent healthy tissue and tumor areas. It is then possible to use $\theta_{}$ to determine the intensity minima representing the background and tumor areas $E_{min}$ and $F_{min}$, using Equation (7). $I_{\theta }$ is the intensity value with a frequency greater than $\theta_{}$. The calculations of $E_{min}$ and $F_{min}$ allow us to make a preliminary localization of the tumor area, where $E_{min}<F_{min}$ is the background and $F_{min}<\sigma$ is the tumor area.

(7) $$E_{min}=min\left(I_{\theta }\right)\;and\;F_{min}=max\left(I_{\theta }\right)$$

Subsequent operations aim to identify tumor regions as significant or low-contrast tumors. To judge the distinguishability and contrast between background and tumor area, we used basic statistical methods to achieve this by comparing standard deviations. If the osteosarcoma is a significant tumor, it is easy to distinguish and segment. Otherwise, as a low-contrast tumor, its localization requires further processing. Standard deviations for background and tumor areas were calculated using Equation (8). In the formula, ∂ represents the intensity, m is the mean value of it, and n represents the number of pixels.

(8) $$S_{j}=\sqrt{\frac{\sum_{}^{}{\left(\partial_{j}-m_{j}\right)}^{2}}{n_{j}}}$$

After determining the initial localization and contrast of the osteosarcoma in the MRI images, we should find the final localization of the osteosarcoma. The final positioning formula is shown in Equation (9).

(9) $$\left\{\begin{array}{l} E\;if\;S_{E}>S_{F}\\ F\;if\;S_{E}<S_{F} \end{array}\right.$$

To enhance the visual appearance of the tumor area, we processed the localized tumor area, ignoring the blank area to make the osteosarcoma area more prominent. First, when the tumor is located in the F region, we use Equation (10).

(10) $$Z_{F}=\frac{I_{F\left(x,y\right)}-F_{min}}{S_{F}}$$

The enhancement method we used also takes into account the updated tumor region minimum. The updated $E_{min}^{\prime }$ and $S_{F^{\prime }}$ of the tumor area are shown in Equations (11) and (12).

(11) $${E^{\prime }}_{{}_{min}}=\frac{1}{x}\sum_{j=E_{min}}^{\sigma }I_{j}$$

In Equation (11), ${E^{\prime }}_{min}$ is the updated minimum value of the tumor area, which is defined as the average intensity value between $E_{min}$ to σ. x represents the pixels in this area.

(12) $$S_{F^{\prime }}=\sqrt{\frac{\sum_{}^{}{\left(\partial_{F^{\prime }}-m_{F^{\prime }}\right)}^{2}}{n_{F^{\prime }}}}$$

In Equation (12), $\sigma_{F^{\prime }}$ represents the intensity value, $m_{F^{\prime }}$ represents the mean valueof the intensity value, and $n_{F^{\prime }}$ represents the number of pixels in the updated tumor area ${F^{\prime }}_{min}$. Moreover, the final calculation is shown in Equation (13) below.

(13) $$Z_{E}=\frac{I_{F\left(x,y\right)}-F_{min}^{\prime }}{S_{F^{\prime }}}$$

Finally, by adding the enhanced image to the preprocessed MRI image, the osteosarcoma localization and enhanced image can be obtained, as shown in Equation (14). $I_{f}$ represents the filtered image. $Z_{i}$ will be defined as $Z_{E}$. or $Z_{F}$ according to Equation (9).

(14) $$Z_{R}=I_{f}+Z_{i}$$

The use of non-parametric localization and enhancement methods can effectively solve the difficulty caused by the osteosarcoma’s complex structure, shape, and location. This method makes the shape of the osteosarcoma clearer, facilitating the subsequent segmentation to obtain more accurate results.

### 3.3. Osteosarcoma Image Segmentation

#### 3.3.1. Deep Feature Aggregation Network (DFANet)

During tumor localization and enhancement, some healthy tissues in the image with close strength values to the osteosarcoma region were localized and enhanced, which forced the use of supervised segmentation techniques to avoid the segmentation of unrelated areas. We used the DFANet model to segment the region of osteosarcoma in the image. DFANet is a very efficient CNN structure with high accuracy, used for semantic segmentation [53].

We improved the Xception network with smaller computational complexity to pursue the inference speed of our proposed method and pre-trained it. Then it is used as the backbone in our model.

#### 3.3.2. Network Architecture

**Figure 6:**
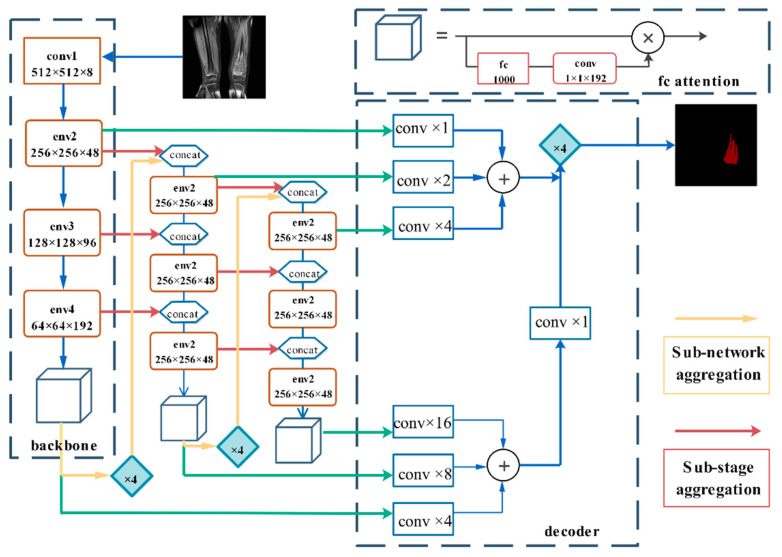
DFANet structure diagram.

The DFANet semantic segmentation network can be regarded as an encoder-decoder structure, as shown in Figure 5. The encoder includes three Xception backlines. Encoders are not only composed of sub-backbone and sub-backbone networks but also some sub-stages that connect this information, combining high-level and low-level characteristics [54]. DFANet implements cross-level feature aggregation. Subnetwork aggregation refers to the up-sampling of advanced feature maps from the previous trunk and the input to the next trunk. Subphase aggregation refers to the transfer of receiving domains and higher-dimensional structural details by grouping layers with the same dimensions [55]. The decoder consists of convolution and bilinear up-sampling operations, which combine the output of each stage to generate segmentation results. Its architecture is shown in Figure 6.

#### 3.3.3. Deep Feature Aggregation

**Figure 7:**
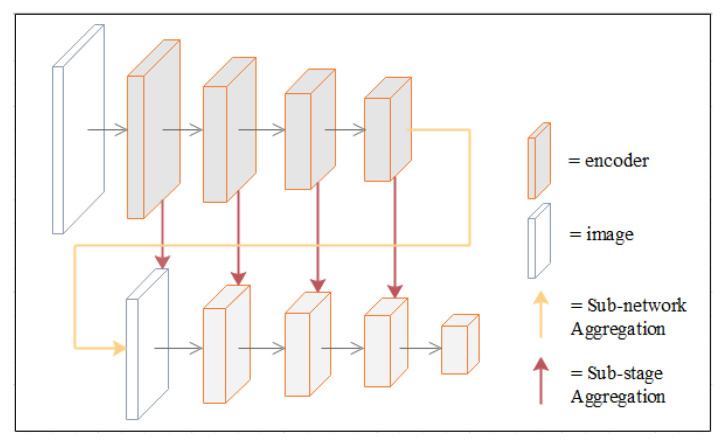
Schematic diagram of deep feature aggregation.

DFANet first learns sub-pixel details by up-sampling the output features of the network and refining the feature map at a larger resolution with another sub-network. To avoid the accuracy loss of high-dimensional features and receptive fields in the feature flow, DFANet achieves stage-level refinement by concatenating feature layers with the same resolution. It reuses high-level features extracted from the backbone network to bridge semantic information and structural details (this refers to the spatial structure, such as edge, shape, etc.). These two aggregation strategies combine detailed and spatial information at different depth locations in the feature extraction network to achieve comparable performance. A schematic diagram of the two aggregation strategies is shown in Figure 7.

Our paper uses a new method for assisted segmentation of osteosarcoma, including denoising and tumor localization and enhancement. It solves the noise and blurred edges of MRI images of osteosarcoma. At the same time, osteosarcoma can be enhanced in complex images, which improves the accuracy and precision of segmentation. The segmentation speed is fast and consumes less memory. The position and range of osteosarcoma can be obtained after the MRI image is processed by the above methods. It provides intuitive image features for subsequent diagnosis and analysis. The value of this method is mainly to provide hospitals and doctors with more accurate auxiliary information for the diagnosis of osteosarcoma. Our method can save resource consumption, including workforce and time costs. If it is promoted clinically, it can effectively improve the current situation of osteosarcoma’s difficult diagnosis.

## 4. Experiments

### 4.1. Dataset

We collected a total of 81,326 clinical images of 204 patients with approximately 400 images per patient, all of which are representative images. Patient-specific information is shown in the table below. We selected about 80% of the data as the training set and about 20% of the data as the test set. Out of 204 patients, 164 training sets and 40 test sets were obtained. Several radiologists and image processing operators participated in the annotation of image data. After identifying the osteosarcoma, they used the itk-snap tool to mark the image. The basic information of patients who provided experimental data is shown in the Table 2.

In addition, to improve the model’s generalization and accuracy, we need to enhance the image data. So we scale, randomly crop, flip horizontally, and rotate the images by 90 degrees and 180 degrees.

### 4.2. Evaluation Indicators

We use some metrics to evaluate the model. Confusion matrices are often used to evaluate network performance in supervised learning and are mainly used to compare classification results with the true classification of instances. Evaluation of our network consists of four parts: the osteosarcoma region predicted by the model as osteosarcoma (True Positive, *TP*), the other regions predicted by the model as osteosarcoma (False Positive, *FP*), areas of osteosarcoma predicted to be non-tumor (False Negative, *FN*), and other areas predicted by the model to be non-tumor (True Negative, *TN*) [56]. The confusion matrix is shown in Table 3 below. According to these four parts, we use the following indicators for evaluation. We use metrics such as accuracy, precision, recall, and F1-score to count the accuracy of segmentation results [57]. We use IOU, DSC to calculate the effect of segmentation based on area. The number of parameters is also used to measure the complexity of the model.

*Acc* is the most commonly used classification performance indicator [58]. It is defined as Equation (18).

(15) $$Acc=\frac{TP+TN}{TP+TN+FP+FN}$$

*Pre* is the percent of all regions predicted to be tumors that are correctly predicted and is defined as Equation (19).

(16) $$Pre=\frac{TP}{TP+FP}$$

*Recall* is the proportion of all true tumor regions that are correctly predicted as tumors and is defined as Equation (20).

(17) $$Re=\frac{TP}{TP+FN}$$

*F*1-score represents the model’s robustness and it is defined as follows [59].

(18) $$F1=2\times \frac{precision\times recall}{precision+recall}$$

*IOU* is the intersection ratio.

(19) $$IOU=\frac{prediction\cap target}{prediction\cup target}$$

*DSC* reflects the similarity of two samples, and it is defined as follows.

(20) $$DSC=2\times \frac{\left|prediction\cap target\right|}{\left|prediction\right|+\left|target\right|}$$

The result of the above metrics can reflect the effect of our segmentation model of osteosarcoma, and we use these metrics to measure model performance.

### 4.3. Comparison Algorithm

We set up comparative experiments to compare our method with FCN [60], PSPNet [61], MSRN [37], MSFCN [62], FPN [63], and U-Net [64] algorithms and analyze the experimental results.

(1) FCN implements pixel-level classification. The problem of repeated storage and calculation of convolutions due to the use of pixel blocks is avoided. This paper uses the FCN-8s and FCN-16s networks, respectively.

(2) PSPNet adopts a pyramid pooling model, which can collect hierarchical information and use global knowledge, promoting the development of scene parsing and semantic segmentation.

(3) MSRN uses residual blocks and convolution blocks to use feature detection at different scales. A simple and efficient reconstruction structure is also designed to easily achieve multi-scale upscaling.

(4) MSFCN uses feature channels to capture contextual information while up-sampling. The accuracy of segmentation is ensured.

(5) FPN adopts a unique feature pyramid model and utilizes the hierarchical semantic features of convolutional networks to achieve feature extraction. It can greatly improve the performance of segmentation models.

(6) U-Net proposes a network structure and a strategy to utilize labeled data efficiently. It uses spliced feature fusion and relies on data enhancement to use data more effectively.

### 4.4. Parameter Setting

The experiments were trained using the “poly“ learning rate strategy, and multiplying the initial rate by (1-maxiteriter) to the power of 0.9, set-ting the basic The learning rate is 2 × 10^−1^. The batch size is 48, and the weight decay is 10^−5^. The cross-entropy error at each pixel on the class is applied as our loss function. All experiments were repeated five times, and the results were averaged. When we train the model, we use three-fold cross-validation to obtain a model with better generalization ability.

### 4.5. Evaluation of Segmentation Effect

**Figure 8:**
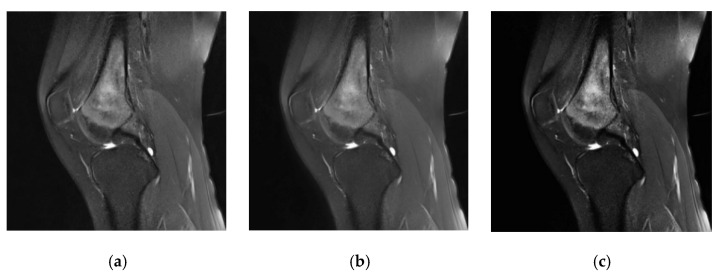
Denoising and tumor localization and enhancement renderings.

In our method, we performed denoising and tumor localization and enhancement on the initial MRI images of the dataset, and the processing effect is shown in Figure 8. The three MRI images of osteosarcoma represent the initial image (Figure 8a), the image after noise removal (Figure 8b), and the image after localization enhancement (Figure 8c). It can be seen that preprocessing can effectively remove noise and enhance the tumor area, which is convenient for improving segmentation accuracy.

**Figure 9:**
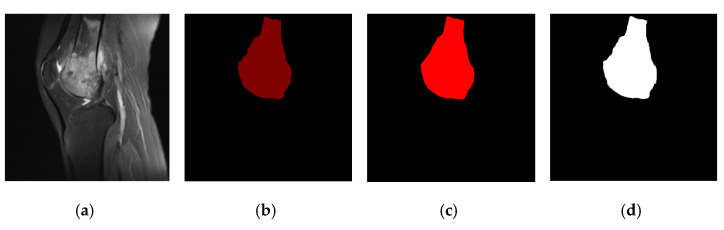
Comparison of segmentation effects before and after preprocessing.

We can see the effect of data processing in Figure 9, including the original osteosarcoma MRI image (Figure 9a), the ground truth (Figure 9b), the segmentation rendering obtained on the unpreprocessed dataset (Figure 9c) and the result after denoising, tumor localization, and enhancement (Figure 9d). We can see the preprocessed model segmentation results are closer to the ground truth, and the edge processing and shape of osteosarcoma are more accurate.

**Figure 10:**
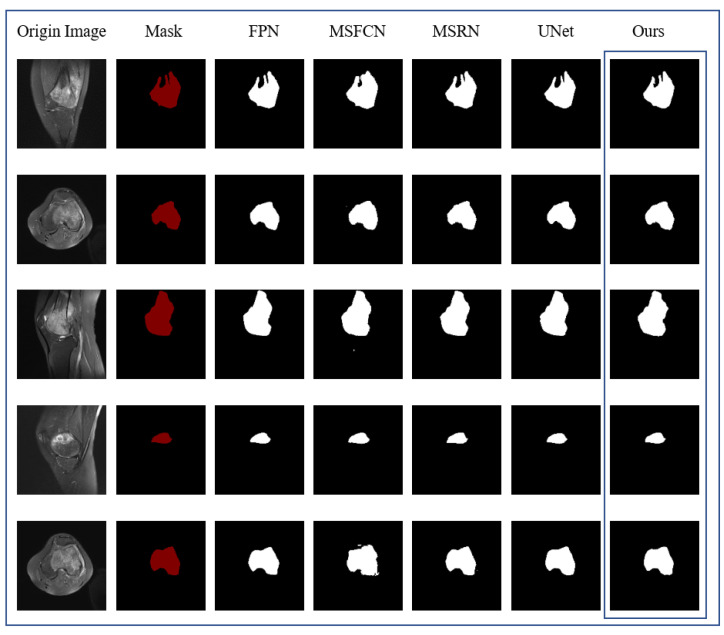
Comparison of segmentation effects of different algorithms.

Figure 10 contains the results of the comparison experiment, showing the intuitive results of the segmentation of the same original MRI image by DFANet and the comparison algorithm, respectively. The first column is the original MRI image of the osteosarcoma before segmentation, and the second column represents the image after marking the position of the osteosarcoma, i.e., the real mask. Columns 3–7 represent the segmentation results of different comparison algorithms, respectively. After comparison, DFANet has better segmentation results. Compared with other algorithms, the images predicted by DFANet are closest to the real labels. The edge processing and predicted shape of osteosarcoma are more accurate.

The performance of our algorithm cannot be wholly determined only by the effect chart. In order to evaluate the effect of the model more accurately, we adopt some indicators to analyze and evaluate the algorithm’s performance. After obtaining the value of each comparison algorithm evaluation index through experiments, it is compared with our method. The result is seen in Table 4. Our method has better performance on each evaluation index. The table also compares the performance indicators of the segmentation effect with or without the data preprocessing operation. It shows that the data set preprocessing effectively improves the segmentation results and optimizes the segmentation boundary. It is necessary to denoise the image data and enhance the tumor area before model training.

**Figure 11:**
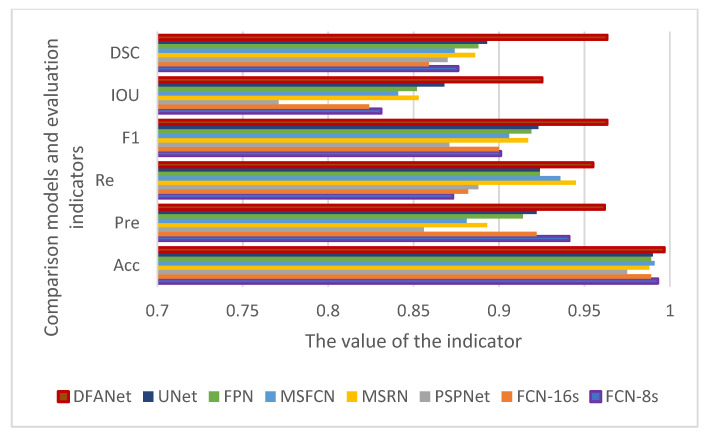
Comparison of evaluation indicators of various algorithms.

In order to compare the effects more intuitively, we drew a line chart as shown in Figure 11. Our model has better performance than other models in each evaluation index. Among them, the *IOU* and *DSC* indicators are nearly 0.1 higher than other models. The *IOU* index is about 0.15 higher than PSPNet, and the precision is nearly 0.1 higher than PSPNet. The F1 indicator is also nearly 0.05 higher than the other models. From various indicators, the indicators of PSPNet are generally the lowest, and the segmentation effect is the worst.

**Figure 12:**
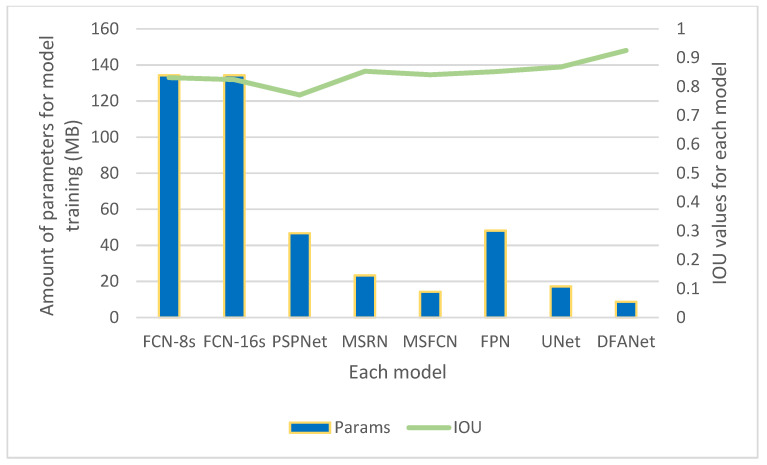
The *IOU* value and the amount of training parameters of the training results of different models.

Figure 12 compares the *IOU* and parameters of DFANet and various comparison algorithms. It can be seen in the image that the *IOU* value of our model is the highest. It also consumes the least number of parameters, only 8.72 M. Among various algorithms, the FCN algorithm has a general segmentation effect but consumes the largest number of parameters. PSPnet has the worst segmentation effect but also consumes a large number of parameters.

**Figure 13:**
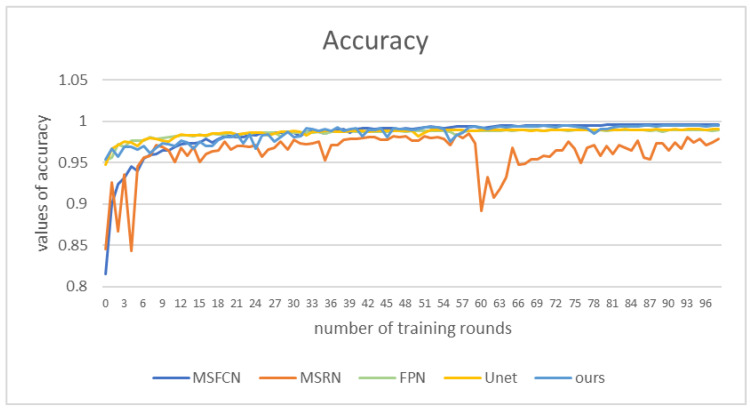
Accuracy values of different models under corresponding epochs.

Figure 13 shows the accuracy values of those methods. Our method’s performance is not as good as that of FPN and UNet in the first 30 rounds of training. However, in the subsequent training, the accuracy value of our model is higher than that of other comparison algorithms, nearly 0.1 higher than MSRN. The performance of MSRN and MSFCN models is very unstable. In contrast, the accuracy value of our model remains above 0.95, and reaches 0.99 in the later stage.

**Figure 14:**
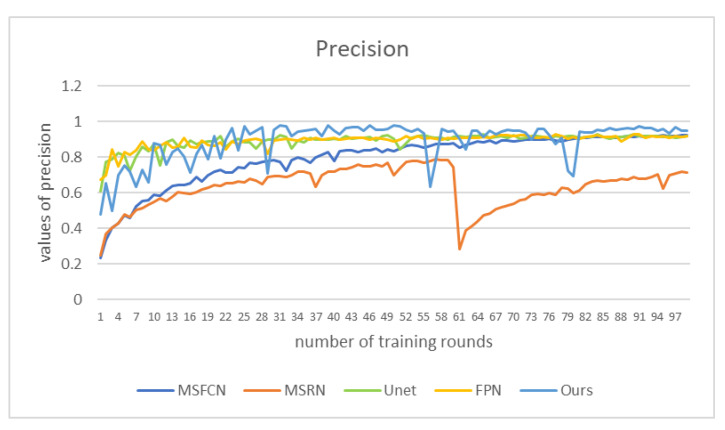
Precision values of different models under corresponding epochs.

We plot the precision as a function of 100 epochs for five of the contrasting models in Figure 14. It can be seen from the figure that the accuracy of our method is the highest most of the time, reaching more than 0.95, which is nearly 0.2 higher than the worst-performing MSRN. Between epochs 30 and 60 and after 80, the precision of DFANet is maintained in a relatively stable and high state, and the obtained segmentation effect is relatively accurate.

**Figure 15:**
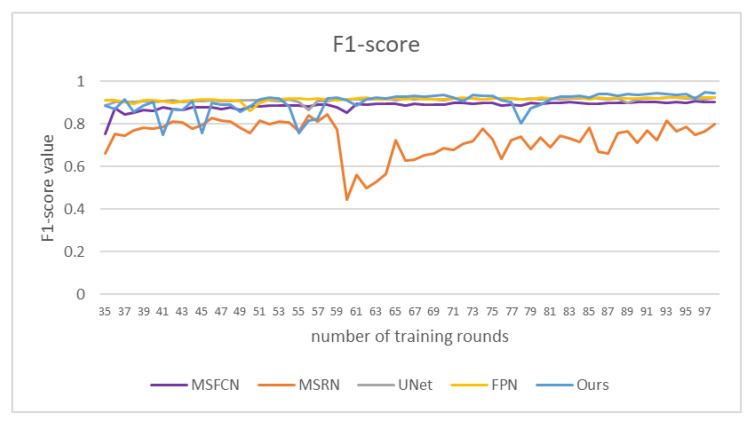
*F*1-score of different models under certain epochs.

We compared the *F*1-score values of different models, as shown in Figure 15. The F1-score value of DFANet gradually increased in the later stage, and reached 0.94 at about 100 rounds of training. Our method has a good segmentation effect and high segmentation accuracy for osteosarcoma.

**Figure 16:**
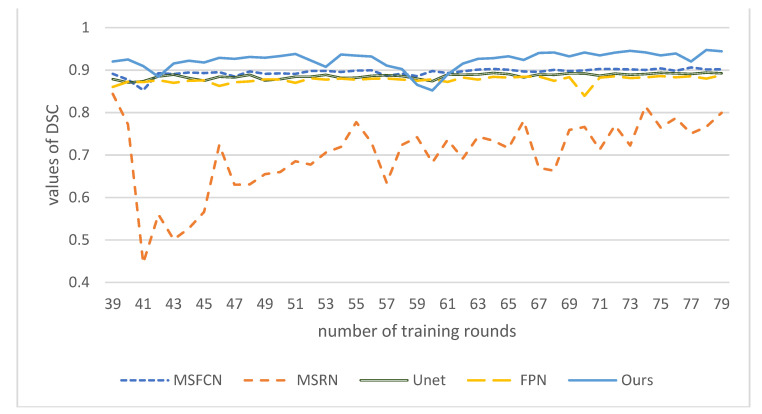
*DSC* values of different models under certain epochs.

Finally, we compared the *DSC* values of these models at corresponding epochs. As shown in Figure 16, the *DSC* value of our model is the highest, close to 0.95, nearly 0.05 higher than MSFCN, and about 0.15 higher than the worst MSRN. *DSC* of our model has a continuous upward trend, and as the number of training rounds increases, its effect will get better and better. It shows that DFANet has high segmentation similarity and can better segment the target area of osteosarcoma and process the boundary.

## 5. Summary

In this paper, an auxiliary segmentation method of osteosarcoma in MRI images based on denoising and local enhancement is proposed. More than 8000 osteosarcoma MRI images collected from hospitals are used as a dataset. We also compare the method with other segmentation models. The results show that our method is very accurate and combines accuracy and speed, reducing the consumption of resources and costs. Using this method to process MRI images of osteosarcoma is helpful for doctors to diagnose patient conditions more efficiently and accurately.

Our method only uses MRI image processing to aid in diagnosing osteosarcoma. In the future, we will work on combining more data sources, relying on images for diagnosis, and studying multimodal learning combined with medical records.

## Figures and Tables

**Table 1:** Some symbols and their meanings.

Symbol	Paraphrase
$P_{n}$	W-output of MSA module
$Q_{n}$	L eFF module
t	MRI image of osteosarcoma with noise
c	clean MRI images
r	residual noise
E	Background regions in MRI images
F	tumor area in the image

**Table 2:** Patient information providing experimental data.

Characteristics		Total Training Set	Test Set	Characteristics
Age	<15	48(23.5%)	38(23.2%)	10(25%)
	15–25	131(64.2%)	107(65.2%)	24(60%)
	>25	25(12.3%)	19(11.6%)	6(15.0%)
Sex	Female	92 (45.1%)	69 (42.1%)	23 (57.5%)
	Male	112 (54.9%)	95 (57.9%)	17 (42.5%)
Marital status	Married	32 (15.7%)	19 (11.6%)	13 (32.5%)
	Unmarried	172 (84.3%)	145 (88.4%)	27 (67.5%)
SES	Low SES	78 (38.2%)	66 (40.2%)	12 (30.0%)
	High SES	126 (61.8%)	98 (59.8%)	28 (70.0%)
Surgery	Yes	181 (88.8%)	146 (89.0%)	35 (87.5%)
	No	23 (11.2%)	18 (11.0%)	5 (12.5%)
Grade	Low grade	41 (20.1%)	15 (9.1%)	26 (65%)
	High grade	163 (79.9%)	149 (90.9%)	14 (35%)
Location	Axial	29 (14.2%)	21 (12.8%)	8 (20%)
	Extremity	138 (67.7%)	109 (66.5%)	29 (72.5%)
	Other	37 (18.1%)	34 (20.7%)	3 (7.5%)

**Table 3:** Patient information providing experimental data.

	Predicted: NO	Predicted: YES
Actual: NO	*TN*	*FN*
Actual: YES	*FP*	*TP*

**Table 4:** Comparison of osteosarcoma segmentation performance of different algorithms.

Model	*Acc*	*Pre*	*Re*	*F*1	*IOU*	*DSC*	Params
FCN-8s	0.993	0.941	0.873	0.901	0.831	0.876	134.3 M
FCN-16s	0.989	0.922	0.882	0.900	0.824	0.859	134.3 M
PSPNet	0.975	0.856	0.888	0.871	0.771	0.870	46.70 M
MSRN	0.988	0.893	0.945	0.917	0.853	0.886	23.38 M
MSFCN	0.991	0.881	0.936	0.906	0.841	0.874	14.27 M
FPN	0.989	0.914	0.924	0.919	0.852	0.888	48.20 M
UNet	0.990	0.922	0.924	0.923	0.868	0.893	17.26 M
Our (DFANet)	0.991	0.943	0.952	0.950	0.903	0.949	11.25 M
Our (Eformer + DFANet)	0.995	0.959	0.955	0.961	0.928	0.964	19.97 M

## Data Availability

Data used to support the findings of this study are currently under embargo while the research findings are commercialized. Requests for data, 12 months after publication of this article, will be considered by the corresponding author.
